# Ensemble of a subset of *k*NN classifiers

**DOI:** 10.1007/s11634-015-0227-5

**Published:** 2016-01-22

**Authors:** Asma Gul, Aris Perperoglou, Zardad Khan, Osama Mahmoud, Miftahuddin Miftahuddin, Werner Adler, Berthold Lausen

**Affiliations:** 1 Department of Mathematical Sciences, University of Essex, Colchester, CO4 3SQ UK; 2 Department of Statistics, Shaheed Benazir Bhutto Women University, Peshawar, Pakistan; 3 Department of Statistics, Abdul Wali Khan University, Mardan, Pakistan; 4 Institute of Medical Informatics, Biometry and Epidemiology, University of Erlangen-Nuremberg, Erlangen, Germany

**Keywords:** Ensemble methods, Bagging, Nearest neighbour classifier, Non-informative features, 62H30, 68T05, 68T10

## Abstract

Combining multiple classifiers, known as ensemble methods, can give substantial improvement in prediction performance of learning algorithms especially in the presence of non-informative features in the data sets. We propose an ensemble of subset of *k*NN classifiers, ES*k*NN, for classification task in two steps. Firstly, we choose classifiers based upon their individual performance using the out-of-sample accuracy. The selected classifiers are then combined sequentially starting from the best model and assessed for collective performance on a validation data set. We use bench mark data sets with their original and some added non-informative features for the evaluation of our method. The results are compared with usual *k*NN, bagged *k*NN, random *k*NN, multiple feature subset method, random forest and support vector machines. Our experimental comparisons on benchmark classification problems and simulated data sets reveal that the proposed ensemble gives better classification performance than the usual *k*NN and its ensembles, and performs comparable to random forest and support vector machines.

## Introduction

In supervised classification tasks, the aim is to construct a predictor that assigns a class label to new observations. To do so the training data is utilized, where a class label is associated with each pattern. The class label of an observation is described by a feature vector. However, in many real life classification problems, one often encounters with imprecise data including non-informative features which dramatically increases the classification error of the algorithms (Nettleton et al. 2010).

To overcome this problem feature selection methods are usually recommended before classification to mitigate the effect of such non-informative features (Liu et al. 2014; Mahmoud et al. 2014). These methods investigate the most discriminative features subset from the original features that increases classification performance of a classifier. However, different feature selection methods will result in different feature subsets for the same data set thus varying feature relevancy. This encourages combining the results of several best feature subsets.

Combining multiple classifiers, known as ensemble techniques, have emerged as promising methods to improve the classification performance of weak learners and have gained a lot of interest in the last two decades (Barandela et al. 2013; Bauer and Kohavi 1999; Maclin and Opitz 2011; Melville et al. 2004). These techniques lead to substantial reduction in classification error in many real life applications and, in general, are more resilient to non-informative features in the data than using an individual model (Khoshgoftaar et al. 2011; Melville et al. 2004). One of the simplest ensemble technique is bootstrap aggregation (bagging), that combines the outputs of classifiers constructed on randomly-generated bootstrap training sets (Breiman 1996a). In bagging, *B* bootstrap samples are randomly drawn from the learning set, and a base learner is developed on each of these samples. A new observation is then classified by majority voting of these individual classifiers. Bagging has been used with numerous variations in the literature (Bauer and Kohavi 1999; Hothorn and Lausen 2003a, b). It is demonstrated that bagging can be used to improve the prediction accuracy of weak classifiers, such as decision trees (Breiman 1996a; Hothorn et al. 2004; Hothorn and Lausen 2005).

One of the simplest and oldest methods for classification is the *k* nearest neighbours (*k*NN) classifier. It classifies an unknown observation to the class of majority among its *k* nearest neighbours observations, as measured by a distance metric, in the training data (Cover and Hart 1967; Guvenir and Akkus 1997). Despite its simplicity, *k*NN gives competitive results and in some cases even outperforms other complex learning algorithms. However, *k*NN is affected by non-informative features in the data, often the case with high dimensional data. Attempts have been made to improve the performance of nearest neighbours classifier by ensemble techniques. Some related work on ensemble of *k*NN classifiers can be found in Grabowski (2002), Domeniconi and Yan (2004), Zhou and Yu (2005), Hall and Samworth (2005) and Samworth (2012).

An ensemble of nearest neighbour classifiers where each member classifier of the ensemble has access to a random feature subset only and the outcomes of these multiple nearest neighbour classifiers are combined for final decision is proposed in Bay (1998). A similar approach based on random feature subsets, random *k*NN based on the idea of random forest, is proposed for classification of high dimensional data sets (Li et al. 2011). Li et al. (2011) rank the features according to their importance and get a final set of features for the final model.

In this manuscript we suggest an ensemble of subset of *k*NN classifiers (ES*k*NN) particularly to deal with the issue of non-informative features in a data set. We applied ES*k*NN to a benchmark and simulated classification problems and compare the results with those of simple *k*NN, bagged *k*NN (B*k*NN), random *k*NN (R*k*NN), ensemble based on multiple feature subset method (MFS), random forest (RF) and support vector machines (SVM). Experiments are carried out on the data sets with their original features set and with some added non-informative features.

## Ensemble of subset of *k*NN classifiers

Let ${\mathcal {L}}={(\mathbf x _i,y_i), i=1 \ldots n}$ be a training set consisting of *n* independent observations, where $\mathbf{x _i}= (x_{i1}, x_{i2}, \ldots , x_{id})$ is a *d*-dimensional feature vector and *y* is the vector of class labels; where ${ y_i \in \{{1, \ldots , J}}\}$, *J* being the total number of classes, here we consider the two class problem, thus ${ y_i \in \{{1,2}}\}$. Based on this available data set ${\mathcal {L}}$, a classifier predicts the class label for a new/test observation with feature vector $(\mathbf {x}^\prime )$. Divide the training data ${\mathcal {L}}$ in two parts, ${\mathcal {L}_{T}}$ and ${\mathcal {L}_{\textit{V}}}$, the first one for construction of the classifiers and the other part for validation. For simplicity we denote the set used for construction of the models ${\mathcal {L}_{T}}$ by ${\mathcal {L} ^ *}$. Let us denote the *d* input features in ${\mathcal {L} ^ *}$ by ${\mathbf {P}} = (p_1,p_2,p_3, \ldots , p_d)$. For a given subset size, say *l*, where $l < d$, a random subset of features ${\mathbf {P}}^{l}$, is drawn from $\mathbf {P}$. Based on the randomly selected features a bootstrap sample is drawn from ${\mathcal {L} ^ *}$. The new bootstrap learning set ${\mathcal {L}^*}^{(l)}$, consists of *l* dimensional feature vector. This process is repeated until we get *m* training sets, ${\mathcal {L}^ *}^{(1l)}, \ldots , {\mathcal {L}^ *}^{(ml)}$, each of $n\times {l}+1$ dimensions. The base *k*NN classifier is constructed on these bootstrap training sets and a set of *m* classifiers is generated.

While, drawing a random sample of the same size *n* from the training set, approximately $\frac{1}{3}$ of the observations are left out from that sample. These observations are called out-of-bag (OOB) observations, and can be utilized for estimation of the classification error (Breiman 1996b). In our framework we use the OOB sample for the assessment of the classifier. The *m* classifiers are then ranked according to their individual classification accuracy on the OOB sample and the first *h* of the *m* classifiers are selected from them. The selected classifiers are then assessed for their collective contribution as an ensemble on the validation set ${\mathcal {L}_{\textit{V}}}$. This is done by starting from the best one among *h* classifiers and then adding one by one the rest of the classifiers to the ensemble.

The formation of the ensemble of subset of *k*NN classifiers can be summarized as:

1. Draw a random sample of size $l < d$, without replacement, of features from the feature vector $\mathbf {P}$ of ${\mathcal {L}^*}$, denote the feature vector by $\mathbf {P}^{l}$.
2. Based on the selected random feature subset $\mathbf {P}^{l}$, draw a random sample of size *n*, ${\mathcal {L}^*}^{(l)}$, from ${\mathcal {L}^* }$.
3. Construct the *k*NN classifier on ${\mathcal {L}^*}^{(l)}$.
4. Calculate the accuracy of the classifier on the OOB sample using the same feature set as used for its construction.
5. Iterate step (1) to (4) *m* times and rank the *m* classifiers according to their accuracies.
6. Select first *h* classifiers with highest accuracies.

These selected classifiers are further assessed as follows:

- The ensemble is started with combining the second best classifier to the first best classifier, and classification performance is evaluated on the validation set ${\mathcal {L}_{\textit{V}}}$. The ensemble is then grown by adding the third best classifier and the performance is measured, this process is carried out for all the *h* classifiers,
  - let ${{\mathcal {BS}}}^{\langle r-1 \rangle }$ be the Brier score of the ensemble of selected best *k*NN models without the *r*th model and ${{\mathcal {BS}}}^{\langle r \rangle }$ be the Brier score of the ensemble of the best models after including the *r*th model, then *r*th model is selected if
    $$\begin{aligned} {{\mathcal {BS}}}^{\langle r \rangle } < {{\mathcal {BS}}}^{\langle r-1 \rangle }. \end{aligned}$$

The ensemble is formed in a two stage procedure by assessing the models using two different performance measures *misclassification rate* and *Brier score*.

In the *first* stage the classification models are evaluated using the *misclassification rate* (MR) as the performance measure. A classification model is desired to have minimum misclassification rate than others used for a classification task, and thus the classification models with a low misclassification rate are selected.

In the *second stage* of the algorithm the selected models are further evaluated using the *Brier score* as a performance measure. The Brier score measures the difference between the observed state of the outcomes of the test instances and the estimated probabilities that are in turn used to classify new observations using some threshold. Besides the traditional misclassification rate and other metrics, Brier score can also be used to evaluate the predictive performance of a classifier. While using output of the classifier as a basis for decision making, a more detailed evaluation is required; where not only the prediction accuracy of the classifier should be considered but also the quality of the estimate needs ample consideration. That can be done through a score such as the Brier score that, in principle, measures the predictive ability/quality of a classifier in classifying new data (Hernández-Orallo et al. 2012; Steyerberg et al. 2010; Kruppa et al. 2014).

Let the class labels of the test instances from the two classes, “positive” and “negative”, are represented by 0, or 1, i.e ${ y \in \{{0,1}}\}$. The Brier score for the probabilities of the predicted class 1, $y=1$, is:

$$\begin{aligned} \mathcal {BS}= & {} E(y_i-p(y_i=1))^2. \end{aligned}$$

An estimator for the above score is:

$$\begin{aligned} \hat{\mathcal {BS}} = \frac{\sum _{i=1}^{n_{t}}\left( y_i-\hat{p}(y_i| \mathbf{{x}})\right) ^2}{n_{t}}, \end{aligned}$$

where, $n_t$ is the total number of test points and the state of the outcome is, ${ y \in \{{0,1}}\}$. A low Brier score indicates better performance of the predictor. Thus the models minimizing the Brier score of the ensemble are selected.

One technical reason for assessing the individually selected models, in the first stage, for their collective contribution using the Brier score is that this score is more capable of determining the contribution of a model, to be included in the ensemble, than the misclassification rate. To illustrate this, let the estimated probability of a test observation belonging to class 1, provided that class 1 is the true class, by a classifier *c*1 is given as:

$$\begin{aligned} \hat{f}_{c1}= 0.56. \end{aligned}$$

Suppose that the cut-off for assigning this observation to class 1 is

$$\begin{aligned} \hat{f}(.) > 0.5, \end{aligned}$$

which implies that the given observation belongs to class 1 and classification error will be 0 (correct classification). The Brier score in this case is 0.1936.

Now consider that the second classifier gives the estimated probability for that observation as 0.68. The combined probability estimate of the two classifiers for the same observation, denoted by $\hat{f}_{{c1,c2}}$, is given as:

$$\begin{aligned} \hat{f}_{{c1,c2}} = 0.62 \ . \end{aligned}$$

Consequently, the Brier score decreases to 0.1444. The classification error in both the cases is 0 as that of a single classifier for the given cut-off.

A third classifier has an estimated probability of 0.88, the resultant combined probability is:

$$\begin{aligned} \hat{f}_{{c1,c2,c3}}= 0.71 . \end{aligned}$$

Here the Brier score decreases to 0.0841 while the classification error remains the same (0) as the previous ensemble of two classifiers for the given cut-off.

This follows that if classification errors are considered for classifier addition into the ensemble, classifier c2 and c3 would not be part of the ensemble, as the error remains the same, whereas the Brier score reduces with the addition of classifiers c1 and c2 thus leading to an ensemble of size 3.

The general pseudo code of ES*k*NN is given in Algorithm 1.

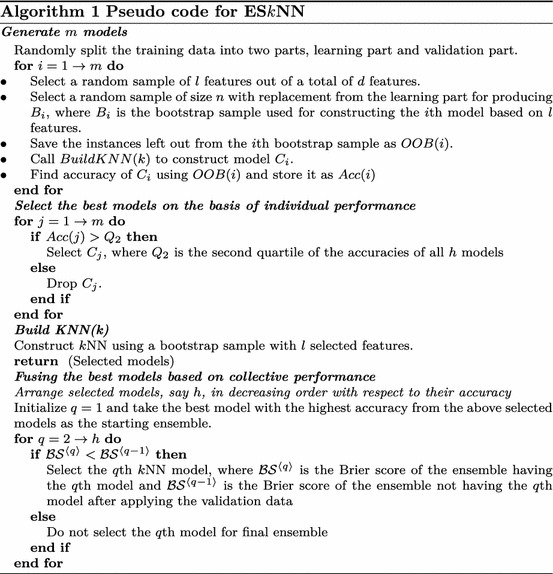



## Simulation study

In addition to bench mark data sets we assessed ES*k*NN by simulation setups. We state two simulation models to assess the performance of ES*k*NN. The models proposed in our simulation study involve several variations to get an understanding of the behaviour of classifiers under different situations. The details of the two models are given below.

### Simulation model 1

In this model, the performance of the classifiers is investigated in different setups. Firstly, the predictors of the two classes are generated with correlated and uncorrelated structures respectively. The variables for class 1 are correlated and generated with a varying variance covariance structure, while the features determining class 2 are independent. A total of 500 independent binary class data sets are generated, each with 20 features. The variables for class 1 are generated from ${\mathcal {N}(2,w\Psi )}$, while those of class 2 generated from ${\mathcal {N}({1},{1})}$. The values considered for *w* in class 1 are 3, 5, 10, 15 and 20. The predictive performance of the algorithms are investigated by adding 50, 100, 200 and 500 non-informative features, generated from normal distribution, to the data. The variance covariance matrix $\Psi$, which is a $d\times {d}$ matrix, is:

1 $$\begin{aligned} \Psi = \left( {\begin{array}{l@{\quad }l@{\quad }l@{\quad }l} \sigma _{1,1} &{} \varrho _{1,2} &{} ,\ldots , &{} \varrho _{1,d} \\ \varrho _{2,1} &{} \sigma _{2,2} &{} ,\ldots , &{} \varrho _{2,d} \\ \vdots &{}\vdots &{} \vdots &{} \vdots \\ \varrho _{m,1} &{} \varrho _{d,2} &{} ,\ldots ,&{} \sigma _{d,d} \end{array}} \right) , \end{aligned}$$

where $\varrho _{ij}$ are the covariances given by

2 $$\begin{aligned} \varrho _{ij} =(1/2)^{|{i-j}|}, i,j=1,\ldots ,d. \end{aligned}$$

and $\sigma _{ij}$, on the diagonal of ${\Psi }$, is the variance, $\sigma _{ij}$ = 1 when *w* is 1. Changing the value of *w* results in different degree of correlation between variables. The data is generated in such a manner that the variables within Class 1 are correlated among each other and are exhibiting negligible/no correlation with the features from Class 2.

### Simulation model 2

The second simulation model, model 2, is a four-dimensional model, derived from the model proposed in Mease et al. (2007). A set of 500 independent binary class data sets are generated each consisting of 1000 observations and 4 features. The feature vector $\mathbf {x}$ is a four dimensional random vector uniformly distributed on [0, 100] and the response variable *y* with two outcomes 0 or 1. The class is determined by the distance *r*, the distance of feature vector $\mathbf {x}$ from the central point. The class probabilities given features are:

3 $$\begin{aligned} p({y=1} \mid \mathbf {x})=\left\{ \begin{array}{ll} 1, &{} \quad \hbox {if }{r< 110},\\ \frac{150-r}{140}, &{} \quad \hbox {if } {110\le {r}\le {140}},\\ 0, &{} \quad {otherwise.} \end{array} \right. \end{aligned}$$

The response values are generated from the above distribution using a Bernoulli random number generator. We extend the dimensions of this model by adding 50, 100, 200 and 500 non-informative feature generated from uniform distribution. The data complexity increases with the increase in the number of added non-informative features.

## Simulation results and discussion

The average misclassification rate, from model 1 and model 2, are presented in Tables 1, 2 and 3.

**Table 1:** Misclassification rate of the methods on the data sets with added non-informative features from model 1

Features	*k*NN	B*k*NN	R*k*NN	MFS	RF	SVM	ES*k*NN
20	0.050	0.047	0.046	0.048	0.052	*0.043*	0.044
$20+50$	0.063	0.058	0.062	0.061	0.055	0.055	*0.047*
$20+100$	0.076	0.067	0.071	0.066	0.066	0.057	*0.046*
$20+200$	0.114	0.104	0.089	0.084	0.063	0.065	*0.046*
$20+500$	0.146	0.127	0.142	0.112	0.062	0.084	*0.046*

The first column shows the number of non-informative features added to the data set. Results of the best performing method are highlighted in italics. The value of $w=1$

The results from model 1, in Table 1 indicate that the classification accuracy of ES*k*NN is higher than all the other methods on most of the cases except for the data with original 20 features where SVM outperforms all the methods. The table reveals that unsurprisingly, *k*NN shows high error rate compared to other methods and the performance of *k*NN based methods declines with the increasing number of non-informative features in the data where as ES*k*NN still perform better. In case of the data set with original features SVM performs better, by giving minimum misclassification rate, as compared to all the other methods.

**Table 2:** Misclassification rate of the classifiers on the data sets from model 1 for different values of *w*, on 70 features ($20+50$ noninformative), listed in column 1

w	*k*NN	B*k*NN	R*k*NN	MFS	RF	SVM	ES*k*NN
3	0.198	0.196	0.185	0.168	*0.084*	0.103	0.147
5	0.221	0.213	0.182	0.169	*0.058*	0.115	0.162
10	0.225	0.198	0.114	0.104	*0.026*	0.100	0.114
15	0.200	0.180	0.057	0.061	*0.012*	0.086	0.076
20	0.185	0.164	0.035	0.041	*0.008*	0.077	0.039

Results of best performing methods for the corresponding value of *w* is shown in italics

**Table 3:** Misclassification rate of the methods on the data sets with added non-informative features from model 2

Features	*k*NN	B*k*NN	R*k*NN	MFS	RF	SVM	ES*k*NN
4	0.125	0.122	0.169	0.122	0.159	*0.101*	0.119
$4+50$	0.170	0.170	0.175	0.169	0.193	0.164	*0.163*
$4+100$	0.194	0.187	0.185	0.205	0.203	0.205	*0.164*
$4+200$	0.242	0.232	0.201	0.216	0.199	0.443	*0.175*
$4+500$	0.276	0.269	0.231	0.249	0.211	0.524	*0.191*

The first column shows the number of non-informative features added to the data set. Results of the best performing method is shown in italic font

From Table 2, there is an increase of misclassification rate of all the classifiers, except random forest. It can be observed that the prediction performance of the *k*NN based classification methods and SVM decrease with high variance and covariance of the data, i.e., for increasing values of *w*. However random forest gives better classification accuracy in this case. Although the performance of *k*NN based methods declines, ES*k*NN consistently perform better than the other methods except from random forest in such situations.

**Fig. 1:**
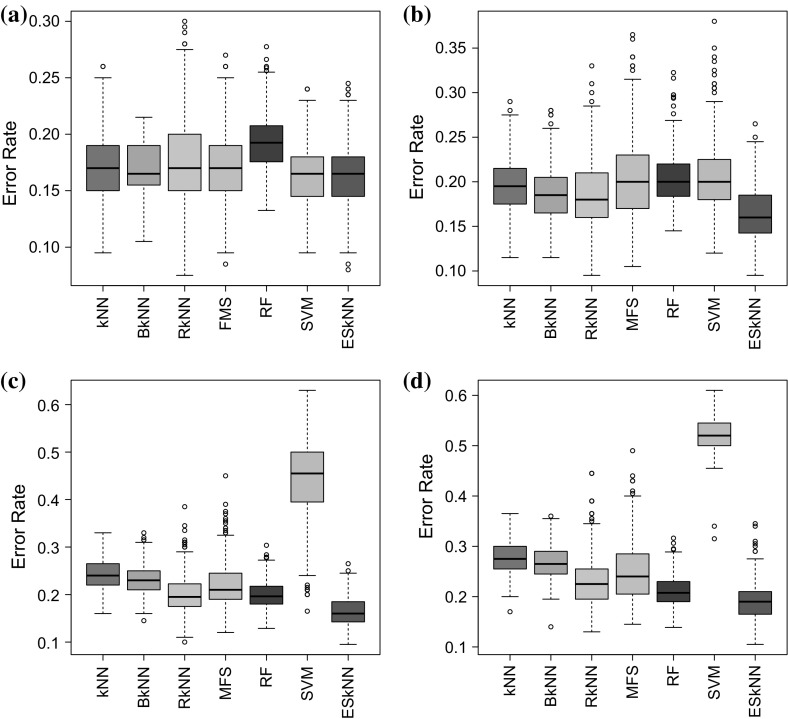
Misclassification rate, of simulated data from model 2 with added non-informative features. **a** 50 added non-informative features; **b** 100 added non-informative features; **c** 200 added non-informative features; **d** 500 added non-informative features

The results of model 2 from Table 3 reveal that ES*k*NN consistently outperform the other methods in the presence of non-informative features in the data, however, in the case of data with original features only, SVM is giving the best result and in case of 100 features ES*k*NN gives better results than other methods and comparable to SVM. Bagged *k*NN provide same results as usual *k*NN on the data with 4 features and slight accuracy gain is achieved than the usual *k*NN on the data with added no-informative features (Fig. 1).

## Experiments on bench mark data sets

The performance of the proposed method in terms of misclassification rate, is evaluated on a total of 31 benchmark data sets. The data sets chosen include a wide range of domain that is microarray gene expression data sets, data sets from life science, finance and physical science. “Diabetes” and “Sonar”, data sets are from R-packages “mlbench” (Leisch and Dimitriadou 2010); ‘dystrophy” and “Glaucoma” are from “ipred” (Peters and Hothorn 2012). All the other data sets are from UCI (Bache and Lichman 2013). Summary of the data sets is given in Table 4.

**Table 4:** Summary of the data sets

Data sets	Sample size	Features	Feature type (continuous/discrete/categorical)
Haberman	306	3	(0/3/0)
Dystrophy	164	5	(2/3/0)
Mammographic	830	5	(0/5/0)
Transfusion	748	5	(2/3/0)
Phoneme	1000	5	(5/0/0)
Bupa	345	6	(1/5/0)
Appendicitis	106	7	(7/0/0)
Diabetes	768	8	(8/0/0)
Biopsy	683	9	(0/9/0)
SAheart	462	9	(5/3/1)
Indian liver	579	10	(5/4/1)
Solar-Flare	322	12	(0/10/2)
Credit approval	690	15	(2/13/0)
House vote	232	17	(0/0/17)
Bands	365	19	(13/6/0)
Hepatitis	80	19	(2/17/0)
Two norms	1000	20	(20/0/0)
German credit	1000	20	(0/7/13)
Body	507	24	(24/0/0)
WPBC	194	33	(31/2/0)
Sonar	208	60	(60/0/0)
Glaucoma	196	61	(61/0/0)
Musk	476	166	(0/166/0)

Number of observations, features and feature type. The first 8 are microarray data sets, the rest are from life, finance, physical, and social science

**Table 5:** Misclassification rate of *k*NN, R*k*NN, B*k*NN, MFS, RF, SVM and ES*k*NN

Data sets	*k*NN	B*k*NN	R*k*NN	MFS	RF	SVM	ES*k*NN
Haberman	0.243	0.24	0.255	0.241	0.271	0.325	*0.237*
Dystrophy	0.117	0.118	0.121	0.110	0.115	*0.099*	0.105
Mammographic	0.190	0.193	0.178	0.183	*0.167*	0.191	0.174
Transfusion	0.233	0.235	0.23	0.225	0.217	0.317	0.218
Phoneme	0.167	0.184	0.171	0.174	0.145	0.204	*0.132*
Bupa	0.320	0.327	*0.219*	0.327	0.271	0.319	0.319
Appendicitis	0.142	0.139	0.144	0.149	0.145	0.224	*0.128*
Diabetes	0.264	0.259	0.263	0.262	*0.233*	0.27	0.256
Biopsy	0.032	0.0311	0.028	0.039	0.027	0.058	*0.020*
SAheart	0.336	0.334	0.343	0.337	*0.289*	0.307	0.317
Indian liver	0.314	0.320	0.290	0.312	0.293	0.373	*0.286*
Solar-flare	0.027	0.026	0.025	0.026	0.025	0.042	*0.022*
Credit Approval	0.319	0.317	0.336	0.194	*0.123*	0.142	0.166
House Vote	0.082	0.082	0.089	0.072	0.036	*0.033*	0.042
Bands	0.389	0.393	0.342	0.383	*0.265*	0.367	0.350
Hepatitis	0.423	0.372	0.288	0.362	0.276	*0.146*	0.321
Two Norms	0.040	0.039	0.029	0.036	0.04	*0.026*	0.033
German Credit	0.307	0.306	0.296	0.308	*0.23*	0.291	0.286
Body	0.023	0.024	0.036	0.025	0.037	*0.016*	0.020
WPBC	0.241	0.240	0.235	0.244	*0.196*	0.285	0.235
Sonar	0.179	0.179	0.157	0.189	0.161	0.169	*0.147*
Glaucoma	0.193	0.193	0.192	0.196	*0.105*	0.122	0.176
Musk	0.142	0.142	0.113	0.114	0.110	0.133	*0.103*

The results of best performing methods on the corresponding data set are highlighted in italics

### Experimental setup

The performance of the ES*k*NN is evaluated on a total of 23 data sets. The ES*k*NN is evaluated in two scenarios on benchmark data sets; in case of benchmark data sets with their original features and then adding non-informative features to the data sets. The performance of ES*k*NN in terms of misclassification rate is compared with usual *k*NN, bagged *k*NN, random *k*NN, MFS, random forest and SVM. Each data set is divided into test and training sets, 90 % of the total data is used for the training and 10 % for testing. The same test and training set is used for all the methods and the results are averaged over a total of 1000 such splits. All the experiments are carried out using R (R Core Team 2013). The value of *k* for $k=1,\ldots ,10$, is selected by tenfold cross validation using the R-Package “e1071” for the *k*NN based methods (Meyer et al. 2012). Random forest is tuned by using R-function “tune.randomForest” available within the same package. For SVM we used “kernlab” R-Package (Karatzoglou et al. 2004). For tuning sigma for SVM, we used the automatic selection available with the “kernlab” R package. The other parameters are fixed at default values. Total of 1001, *k*NN models are generated on bootstrap samples and then 40 % of the total are reselected for the second stage. The number of models generated is taken an odd number to break ties in voting on the classifiers for classification of a test point. The feature subset size is set to one-third of the input features, however, in low dimensions, in case of original features in the data, i.e., $d<6$ the feature subset size is taken as 2.

## Results and discussion

The results on the data sets with their original features and with added 500 randomly generated non-informative features are reported in Tables 5 and 6 respectively. The results from Table 5, show that ES*k*NN outperform or giving comparable results to other methods considered here. It is interesting to note that in case of the data sets with their original features ES*k*NN consistently outperform the *k*NN based methods on most of the data sets and gives comparable results to random forest. ES*k*NN gives overall better results on 8 data sets, on 9 data sets random forest is better than all the methods, on 5 data sets SVM is giving minimum classification error and on one data sets R*k*NN outperforms the rest of the methods.

In case of non-informative features in the data, Table 6, on 11 data sets ES*k*NN gives minimum classification error than the other methods, on 9 data set RF is giving best classification performance and on one data set SVM is giving better results and on two data sets their is no clear winner between random forest and ES*k*NN, however, ES*k*NN gives better performance than *k*NN based methods and SVM. Here again, it is observed that ES*k*NN results in smaller classification error than *k*NN based methods on most of the data sets.

**Table 6:** Misclassification rate of *k*NN, R*k*NN, B*k*NN, MFS, RF, SVM and ES*k*NN with added non-informative features to the data sets

Data sets	*k*NN	B*k*NN	R*k*NN	MFS	RF	SVM	ES*k*NN
Haberman	0.278	0.274	0.279	0.269	0.263	0.429	*0.260*
Dystrophy	0.249	0.248	0.291	0.237	*0.118*	0.252	0.204
Mammographic	0.217	0.223	0.180	0.225	*0.158*	0.527	0.189
Transfusion	0.238	0.237	0.237	0.239	0.236	0.517	*0.230*
Phoneme	0.279	0.279	0.252	0.351	0.269	0.538	*0.243*
Bupa	0.362	0.352	0.389	0.376	0.342	0.560	*0.330*
Appendicitis	0.207	0.209	0.277	0.209	*0.150*	0.215	0.197
Diabetes	0.358	0.354	0.349	0.348	*0.248*	0.530	0.328
Biopsy	0.065	0.067	0.086	0.102	*0.027*	0.067	0.052
SAheart	0.414	0.395	0.349	0.347	0.345	0.509	0.345
Indian liver	0.316	0.315	0.286	0.286	0.286	0.519	*0.275*
Solar-flare	0.027	0.022	0.021	0.025	0.022	0.022	0.022
Credit approval	0.354	0.354	0.320	0.345	0.322	0.546	*0.317*
House vote	0.128	0.125	0.126	0.112	*0.032*	0.109	0.095
Bands	0.405	0.396	0.358	0.354	0.359	0.549	*0.343*
Hepatitis	0.362	0.371	0.380	0.410	0.387	*0.160*	0.333
Two norms	0.047	0.045	0.038	0.052	0.038	0.052	*0.034*
German credit	0.308	0.305	0.301	0.371	*0.285*	0.517	0.300
Body	0.098	0.098	0.099	0.098	*0.049*	0.092	0.088
WPBC	0.262	0.251	0.235	0.235	0.235	0.252	*0.225*
Sonar	0.164	0.164	0.161	0.225	0.242	0.314	*0.156*
Glaucoma	0.256	0.249	0.242	0.272	*0.154*	0.236	0.242
Musk	0.184	0.182	0.169	0.168	0.165	0.290	*0.161*

The results of best performing methods on the corresponding data set are highlighted in italics

## Conclusion and outlook

Considering the idea of ensemble techniques, we have proposed an ensemble of subset of *k*NN classifiers (ES*k*NN) for classification tasks particularly to deal with the issue of non-informative features in the data sets. Our approach consists of forming an ensemble of best *k*NN models thus implicitly digging out the informative features subsets and discarding the non-informative ones. ES*k*NN is assessed for its classification performance on simulated and benchmark data sets. Our results on simulated and benchmark data sets show that the ES*k*NN gives comparable results to RF and outperform *k*NN and *k*NN based ensembles. The results from the simulations, Table 2, reveal that in case of high variance in the classes RF performs better than the others. Random projection ensemble classification (Cannings and Samworth 2015) may allow further improvements. Moreover, it would be of interest to investigate if recent proposals as predictive hubs (Lausser et al. 2014) and representative prototypes (Müssel et al. 2015) can be exploited to develop ES*k*NN further. ES*k*NN is implemented and available as R-Package “ES*k*NN” on CRAN (Gul et al. 2015).
